# Local temperature control improves the accuracy of cardiac output estimation using lung‐to‐finger circulation time after breath holding

**DOI:** 10.14814/phy2.14632

**Published:** 2020-11-07

**Authors:** Tomoyuki Tobushi, Kazuyuki Matsushita, Kouta Funakoshi, Kazuhiro Sakai, Manabu Akamatsu, Yasuko Yoshioka, Takeshi Tohyama, Masayuki Hirose, Ryo Nakamura, Toshiaki Kadokami, Shin‐ichi Ando

**Affiliations:** ^1^ Department of Cardiovascular Medicine Saiseikai Futsukaichi Hospital Chikushino Japan; ^2^ Advanced Industrial Services Fuji Xerox Co., Ltd. Kanagawa Japan; ^3^ Center for Clinical and Translational Research Kyushu University Hospital Fukuoka Japan; ^4^ Imaging Device Development Fuji Xerox Co., Ltd. Kanagawa Japan; ^5^ Sleep Apnea Center Kyushu University Hospital Fukuoka Japan

**Keywords:** cardiac output, heart failure, lung to finger circulation time

## Abstract

As timely measurement of the cardiac index (CI) is one of the key elements in heart failure management, a noninvasive, simple, and inexpensive method of estimating CI is keenly needed. We attempted to develop a new device that can estimate CI from the data of lung‐to‐finger circulation time (LFCT) obtained after a brief breath hold in the awake state. First, we attempted to estimate CI from the LFCT value by utilizing the correlation between 1/LFCT and CI estimated with MRI. Although we could obtain LFCT from 45 of 53 patients with cardiovascular diseases, we could not find the anticipated relation between 1/LFCT and CI. However, we realized that when we adopted only LFCT from patients with a finger temperature of ≥31°C, we could obtain a consistent and clear correlation with CI (correlation coefficient, *r* = .81). Thus, we next measured LFCT before and after warming the forearm. We found that LFCT decreased after the local temperature increased (from 27.5 ± 13.6 to 18.4 ± 5.3 s, p < 0.01). The correlation between the inverse of LFCT and CI improved after warming (1/LFCT vs. CI, from *r* = .69 to *r* = .82). The final Bland–Altman analysis between the measured and estimated CI values revealed that the bias and precision were −0.05 and 0.37 L min^−1^ m^−2^, respectively, and the percentage error was 34.3%. This study clarified that estimating CI using a simple measurement of LFCT is feasible in most patients and a low fingertip temperature strongly affects the CI‐1/LFCT relationship, causing an error that can be corrected by proper local warming.

## Graphical abstract

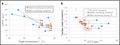

**New & Noteworthy**

Lung‐to‐finger circulation time (LFCT) of blood is well correlated with cardiac index (CI). We developed a new device that measures LFCT after a brief breath hold to simply estimate CI. We obtained LFCT from most patients but found that low finger temperatures (<31°C) substantially increased the time. In a second study, we confirmed that, in patients with low finger temperature, warming the finger to ≥31°C could restore proper LFCT to enable correct CI estimation.

## 1 INTRODUCTION

Because of the advance of cardiac therapies like percutaneous coronary intervention, cardiac surgery or device therapy, more patients were rescued from their critical conditions than in the previous era, though their cardiac function is left depressed. Thus, the therapeutic advances increased the number of patients with heart failure (HF) who have low cardiac function in developed countries. HF is a lifelong chronic condition with a high readmission rate. As the estimated number of patients with HF is 6.2 million in the United States (Virani et al., 2020) and 1 million in Japan (Okura et al., 2008), continuous and efficient management of patients (Yancy et al., 2017) to prevent exacerbation of HF is becoming more important.

Cardiac output (CO) is a clinically important index in HF diagnosis and treatment along with estimation of the degree of pulmonary congestion. The gold standard for obtaining CO has been through heart catheter measurement (Swan et al., 1970); however, this method is highly invasive for repeated daily measurements. Although other methods such as echocardiography and cardiac Magnetic Resonance Imaging (MRI) are also used to measure CO, echocardiographic examination requires high examiner skills and is affected by the chest structure of the patient (Dittmann et al., 1987). MRI requires an expensive machine, and each measurement is costly and each imaging session takes a long time. An ideal device for measuring CO in daily clinical practice should be noninvasive, simple, and inexpensive, thus allowing repeated measurements at the bedside.

The blood circulation time is known to correlate with cardiac function or CO (Chapman & Fraser, 1954; Hall et al., 1996) and has been clinically used for estimating CO until the development of current devices. We have developed an algorithm for the computer‐based automatic estimation of CO using whole night polygraphy data, and demonstrated that cardiac index (CI: CO divided by body surface area values) was strongly associated with lung‐to‐finger circulation time (LFCT), (Dajani et al., 2016; Hosokawa et al., 2015). Recently, a study that used the relationship between LFCT and CI for the noninvasive estimation of CI was published (Orr et al., 2016).

### 1.1 Research purposes

We hypothesized that we would be able to extend the method for the measurement of LFCT in awake patients if a patient can hold breath for a sufficient time to decrease the blood oxygen level adequately below the measurable level. Although there are several methods for the measurement of blood circulation time, they require the injection of some drugs (Conn et al., 1957) or inhalation of gas (Gubner et al., 1939), and each method needs a special device, thus making them unsuitable for daily practice. Hence, we planned to develop a new device that can detect LFCT by measuring the time from the restart of breathing after a certain duration of breath holding to the point at which the saturation of peripheral oxygen (SpO_2_) shows the minimum value and thereafter begins to increase again. Particularly, we aimed to develop a device that can be easily carried by medical personnel and used at the bedside or outpatient clinic.

The purposes of this study were as follows:

To investigate the feasibility and accuracy of LFCT measurement with a simple device using ordered breath hold, we performed two studies.

1. In study 1, we aimed to investigate whether LFCT measured using the new device correlates with the CI measured using a gold standard method, and to determine what factors affect the accuracy of the measurement.
2. In study 2, if some factors could be obtained from study 1, we aimed to create a model using those factors and perform a verification.

## 2 METHODS

We conducted two clinical studies after obtaining approval from the ethics review board of Saiseikai Futsukaichi Hospital. Written informed consent was obtained from all patients.

**FIGURE 1:**
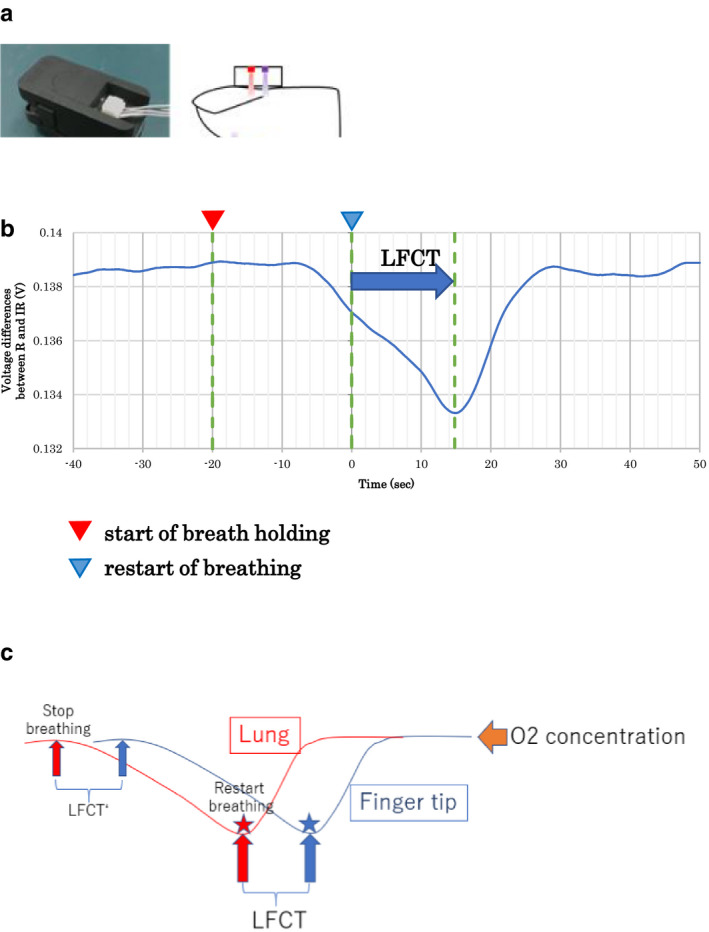
(a) A vertical cavity surface‐emitting laser sensor for setting at the fingertip. The photodiode at the ventral aspect received transmitted light from the dorsal aspect and reflected light from the ventral aspect. From the two‐wavelength transmitted light, a value equivalent to the saturation of peripheral oxygen (SpO_2_) was measured, and the local blood mass and blood velocity were measured from the reflected light. (b) Typical trace during LFCT measurement. Shortly after stopping breathing, the difference in signal voltages of the photodiode light between red light and infrared light (≈SpO_2_) decreased and soon increased again after restarting breathing. The time from the restart of breathing to the minimum value of SpO_2_ was defined as LFCT. In this example, the LFCT value is 14.8 s. (c) We used the difference in times between at the point of stopping of breathing (small red arrow) and the point of starting of SpO_2_ decrease (small blue arrow) or the difference in times between at the point of restarting of breathing (red star) and the point of starting of SpO_2_ increase (nadir: blue star). PD, photodiode; LFCT, lung‐to‐finger circulation time; R, red light; IR, infrared light

A new device equipped with a vertical cavity surface‐emitting laser sensor (Fuji Xerox Co.) was developed and used for the current studies. The device is set at the fingertip similar to a SpO_2_ monitoring device. This device utilizes a two‐wavelength transmitted light (red and infrared light) and a one‐wavelength reflected light (infrared light). From the transmitted light, this device calculates the difference in photodiode signal levels between the direct current (DC) component of the transmitted red light and infrared light, which corresponds to SpO_2_. From the reflected light (infrared light), this device calculates the blood mass and blood velocity at the fingertip to provide coefficients of the CI‐1/LFCT relationship (Figure 1a). This device calculates the difference in the red light and infrared light of the photodiode‐received signal voltage. Usually, oxygen sensors use time average methods to remove high‐frequency artifacts and record stable signals (McClure et al., 2016). The settings of time average are determined by manufacturers and users. Because our device does not use time‐averaging method, our device might theoretically detect the temporal SpO_2_ change more sensitively than the existing SpO_2_ monitors.

### 2.1 LFCT measurement and judgment

We defined LFCT as the time from the start of rebreathing after 20 s of breath holding to the point of the minimum signal voltage difference between the red light and infrared light, which corresponds to the minimum SpO_2_ (Figure 1b). LFCT was measured more than three times, and the data were stored. The suitability of each LFCT waveform for the measurement was examined offline by multiple researchers who were blinded to the patient background, and an average value was calculated for each patient using only the results with a clear peak. Data were excluded from the study when the following conditions were met: (a) no peak value appeared within 50 s after resuming breathing (the minimum value appeared at the time of resuming breathing or at the end of measurement) and (2) multiple peaks appeared and it was not possible to determine one reasonable peak. To clearly determine the respiratory resumption point, we manually decided the points of breath resumption on the individual record afterward referring to all the airflow signals.

As presented in Figure 1b, we tried to measure the lag time of changes in O_2_ concentration in the blood at the Lung and at the Fingertip (LFCT). For this purpose, we can use the difference in times between at the point of stopping of breathing (small red arrow) and the point of starting of SpO_2_ decrease (small blue arrow) or the difference in times between at the point of restarting of breathing (red star) and the point of starting of SpO_2_ increase (nadir: blue star; Figure 1c). Because to measure exactly the ambiguous point of start of decrease in SpO_2_ is practically impossible, we had to use time lag between restart of breathing and comparably sharp nadir of SpO_2_ at fingertip as LFCT.

### 2.2 MRI protocol

In both studies, we measured CI using MRI on the same day. CI was measured using phase‐contrast MRI (PC‐MRI) with a 3.0‐T MRI system equipped with a phased array 32‐channel receiver coil (Ingenia, release 4.1; Philips Healthcare), (Hundley et al., 1995). The PC‐MRI protocol was performed with retrospective electrocardiogram (ECG) triggering and breath holding. To quantify the blood flow of the ascending aorta, the PC‐MRI protocol was applied in the cardiac MRI protocol and the forward blood flow in the ascending aorta was calculated using a standard workstation (Extended MR WorkSpace; Philips Healthcare).

### 2.3 Study 1

#### 2.3.1 Study setting and subjects

The participants were recruited from patients aged >20 years who were admitted to the cardiovascular department from August 2017 to February 2018 regardless of the type of cardiac disease. The exclusion criteria were as follows: requirement for oxygen administration during the test, inability to hold breath for 20 s, inability to provide consent because of dementia, hemodialysis treatment, and contraindications to MRI.

#### 2.3.2 Procedure for LFCT measurement

In the supine position, an air‐flow sensor (TR‐101A, Nihon Kohden Corp.) was attached to each patient's nose to detect the stopping and restarting of breathing, and the new device was fixed at the fingertip. The patients were instructed to hold breath for 20 s, and LFCT was measured more than three times after the measurement of finger temperature using a portable noncontact thermometer (PT‐3S, OPTEX FA CO., LTD). We compared the values with 1/LFCT and CI derived from cardiac MRI conducted on the same day.

Using the data obtained in study 1, we attempted to yield a correction formula including parameters other than LFCT. As a result, we noticed that the temperature of the fingertip may be a major confounding factor for the LFCT measurements. Although the correlation coefficient was improved as shown in Results section when we focused on patients with fingertip temperature ≥31°C, we planned study 2 in order to verify this theory in detail.

### 2.4 Study 2

#### 2.4.1 Study setting and subjects

Study 2 was conducted from September 2018 to October 2018 in patients admitted to the cardiology department, outpatients, and healthy volunteers >20 years old. The exclusion criteria were as follows: requirement for oxygen administration, inability to hold breath for 20 s, a diagnosis of dementia, dialysis treatment, and severe lung disease.

#### 2.4.2 Procedure and measurement

In addition to the three measurements of LFCT, we measured LFCT again after warming the patient's entire forearm and wrist after recording the finger temperature. We covered the patient's one side wrist with a disposable heating pad. After 10 min, we checked the fingertip temperature and proceeded process to measure LFCT. After the measurement, no patients showed burned or other skin disorders on the wrist. The LFCT values obtained before and after warming were each averaged, and the two LFCT values were compared with the CI derived from cardiac MRI obtained on the same day. We measured a tympanic membrane temperature (MC‐510, Omron Healthcare) to record body core temperature before and after warming.

### 2.5 Target sample size

As these studies were exploratory investigations, we set the target sample size from the viewpoint of feasibility. We planned to finish study 1 within a half year, and about 500 new patients were expected to be admitted to our cardiovascular department during the period.

We excluded patients with short‐term hospitalization, those who were unable to follow the breath hold order because of very old age, and those with a severe status precluding breath holding. Moreover, we needed to take into account the capacity of our institution to perform cardiac MRI.

Consequently, we estimated that the possible number of patients during the period would be 40–50. For study 2, as we set the recruitment period at 1 month and we did not consider that a large number of measurements were necessary to prove the hypothesis, we set the target number of patients at 10.

### 2.6 Statistical analysis

In studies 1 and 2, the correlation coefficient was obtained by comparing the CI measured using MRI and the CI estimated from LFCT. Because our previous study (Hosokawa et al., 2015) showed an inversely proportional relationship between 1/LFCT and CI, we analyzed the correlation between 1/LFCT and CI. In study 1, the correlation coefficient was obtained by comparing the CI measured using MRI and 1/LFCT obtained with regression analysis. In study 2, the correlation coefficient was obtained by comparing the CI measured using MRI and the CI calculated using the estimation formula including 1/LFCT and four parameters in regression analysis. In study 1, the parameters were selected using the multivariable analysis stepwise method for the CI estimation correction formula. In study 2, the paired *t*‐test was used for comparison before and after warming, and Bland–Altman plot analysis of the data was performed. Statistical analysis was performed in Excel 2016 (Microsoft Corporation), R version 3.5.1 (R Foundation for Statistical Computing). A *p* < .05 was considered statistically significant. Data are shown as mean ± *SD*.

## 3 RESULTS

### 3.1 Study 1

#### 3.1.1 Patients’ background

A total of 53 patients were enrolled and eight patients were excluded for the following reasons: insufficient breath hold (*n* = 3), no (negative) peak in the LFCT measurement (*n* = 2), severe pulmonary disease (*n* = 2), or inadequate MRI measurement (*n* = 1). The mean patient age was 67.2 ± 12.6 years, and 35 patients (77.8%) were men. The number of patients with HF was 14 (31.1%). The main reasons for admission were ischemic heart disease (*n* = 16, 35.6%) and treatment for arrhythmia (*n* = 9, 20.0%), and other reasons include secondary hypertension, syncope, pulmonary embolization, or rehabilitation after surgery. The data of temperature and heart rhythm are shown in Table 1. We measured LFCT only in the case who could hold breath for 20 s. In the case who could not hold breath, we excluded the unsuccessful session and asked the patient to try again. Finally, however, there were three patients who could not hold breathing for 20 s in all trials, and we judged as “insufficient breath hold” and excluded 3 patients.

**TABLE 1:** Clinical characteristics of subjects of study 1 and 2

	Study 1	Study 2
*n*	45	9
Men (*n*, %)	35 (77.8)	7 (77.8)
Age (years)	67.2 ± 12.6	51.1 ± 20.1
Height (cm)	163.6 ± 8.7	165.6 ± 9.1
Body weight (kg)	64.9 ± 14.3	61.7 ± 9.8
Temperature		
Body temperature (°C)	36.2 ± 0.53	36.2 ± 0.33
Finger temperature (°C)	30.2 ± 2.3	28.4 ± 4.5
Heart rhythm		
Sinus rhythm	37 (82.2)	
Atrial fibrillation (*n* (%))	8 (17.8)	


Values are mean ± *SD*

#### 3.1.2 CI‐1/LFCT relationship and finger temperature

**FIGURE 2:**
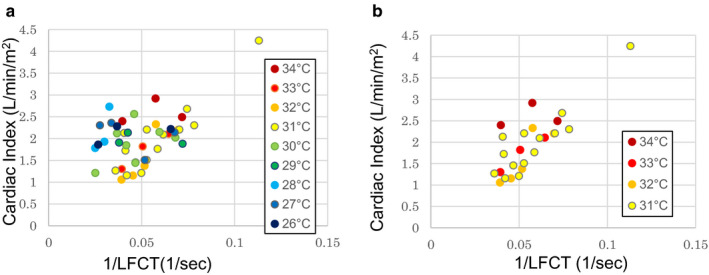
(a) Relationship between 1/LFCT and cardiac index in study 1. The inset shows the finger temperatures. Points are color‐coded according to the finger temperature. (b) Data from only patients with finger temperature ≥31°C showing a clear positive correlation. LFCT, lung‐to‐finger circulation time

The CI‐1/LFCT relationship in the 45 cases obtained in study 1 is shown in Figure 2a. The correlation coefficient between 1/LFCT and CI was as low as *r* = .51 in all 45 cases. Although we expected a negative correlation between 1/LFCT and CI based on previous studies, we could not obtain such a correlation. However, we realized that when we removed the LFCT data of patients whose fingertip temperature was <31°C (*n* = 20), a clear positive correlation was observed between 1/LFCT and CI, with *r* = .81 (Figure 2b). We subanalyzed patients in study 1 dividing into with or without atrial fibrillation rhythm. The subanalysis shows that the atrial fibrillation group (*n* = 8) showed no positive correlation between CI‐1/LFCT like in those without atrial fibrillation (*n* = 37; *r* = .61). Results are shown in Figures S1a and S1b.

#### 3.1.3 Determination of correction factors for CI measurement

To improve the accuracy of the measurement of CI, variables that significantly affected the CI value were selected using a stepwise method. The examined items were as follows: 1/LFCT, age, sex, room temperature, body temperature, fingertip temperature, height, weight, smoking habit, systolic/diastolic blood pressure, pulse rate, resting SpO_2_, postbreathing ΔSpO_2_ desaturation, alternating current (AC) component intensity and DC component intensity of red light, AC component intensity and DC component intensity of infrared light, AC/DC component intensity ratio of red light, AC/DC component intensity ratio of infrared light, fingertip blood mass, and blood flow velocity obtained with our new device.

As a result, we identified four items with the greatest influence (1/LFCT, ΔSpO_2_ after breath holding, AC component intensity of red light [AC_Red_], and AC/DC component intensity of infrared light [AC/DC_IR_]). The estimation formula was set as follows:

$$\text{estimate}\;\text{CI}\;=\;a+\frac{b}{\text{LFCT}}+c*\Delta {\text{SpO}}_{2}+d*{\text{AC}}_{\text{Red}}+e*\text{AC}/{\text{DC}}_{\text{IR}}$$

(a=0.54,b=21.84,c=0.10,d=‐0.08,e=0.01)

$$\text{Adjusted}\;\text{coefficient}\;\text{of}\;\text{this}\;\text{equation}\;\text{was}\;r^{2}=.83.$$

However, as we could not identify the influence of fingertip temperature with this method and the influence of temperature was considered overwhelming compared with the influence of other factors, we decided to perform actual warming of the forearm and wrist to increase the fingertip temperature.

### 3.2 Study 2

#### 3.2.1 Subjects’ background

We recruited 10 subjects but excluded 1 subject who showed unstable LFCT values. The mean age was 51.1 ± 20.1 years, and seven patients (77.8%) were men. Four patients (44.4%) had HF.

#### 3.2.2 Effect of warming on LFCT value

**FIGURE 3:**
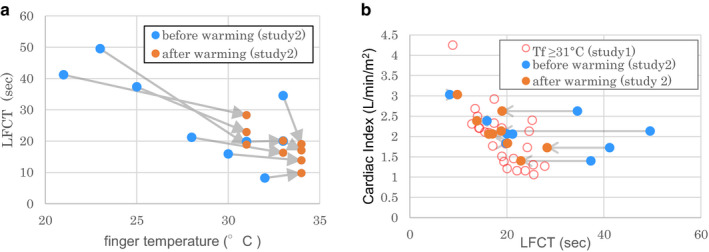
(a) Changes in the relationship of LFCT to finger temperature before and after warming. Seven of nine cases showed decreased LFCT with increased finger temperature. In both of the remaining two cases, the finger temperatures were ≥31°C before warming and the LFCT changes were small. (b) Relationship between LFCT and cardiac index from the data of those with finger temperature ≥31°C in study 1, and data before and after warming in study 2. The arrows show that by warming, the data in study 2 became close to those from patients with finger temperature ≥31°C in study 1. LFCT, lung‐to‐finger circulation time; Tf, finger temperature

By warming, the fingertip temperature was increased and the LFCT values were decreased, as shown in Figure 3a. Only two subjects showed increased LFCTs after warming, although the finger temperature was 32 and 31°C, respectively, before warming. In these cases, the finger temperature increased to 34 and 33°C, respectively, whereas the mean LFCTs increased from 8.2 to 9.8 and 19.8 to 20.1 s, respectively. Overall, the mean finger temperature increased from 28.4 ± 4.5°C to 32.7 ± 1.3°C (*p* < .01) by warming and LFCT decreased from 27.5 ± 13.6 to 18.4 ± 5.3 s (*p* < .01). The tympanic temperatures increased minimally (temperature change 0.13 ± 0.16°C, *p* = .05, maximal elevation was 0.5°C).

Dots representing patients with finger temperatures ≥31°C (*n* = 25) from study 1 and dots representing all subjects after warming from study 2 (*n* = 9) were plotted in an overlaid format in Figure 3b. Here, we found that the dots before warming in study 2 became close to the line for patients with finger temperatures ≥31°C from study 1.

#### 3.2.3 Change in the CI‐1/LFCT relationship by warming and Bland–Altman plot analysis

**FIGURE 4:**
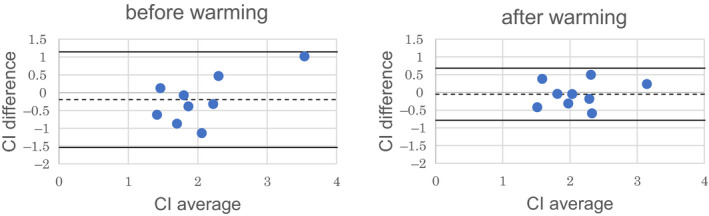
Bland–Altman plots before and after warming in study 2. We analyzed the relationship between the CI measured with MRI and the estimated CI calculated from LFCT before and after warming, by applying the following formula: $\text{estimate}\;\text{CI}\;=\;a+\frac{b}{\text{LFCT}}+c*\Delta {\text{SpO}}_{2}+d*{\text{AC}}_{\text{Red}}+e*\text{AC}/{\text{DC}}_{\text{IR}}$ (*a* = 0.54, *b* = 21.84, c = 0.10, *d* = −0.08, *e* = 0.01). Before warming: The bias and precision were −0.20 and 0.67 L min^−1^ m^−2^, respectively. The percentage error was 62.7%. After warming: The bias and precision were −0.05 and 0.37 L min^−1^ m^−2^, respectively. The percentage error was 34.3%. Solid line: bias. Dashed lines: limits of agreement. CI, cardiac index; LFCT, lung‐to‐finger circulation time

When we compared the CI‐1/LFCT correlation before and after local warming, the correlation between CI and inversed of LFCT significantly improved from 0.69 to 0.82. Bland–Altman analysis of the agreement between the measured CI and the CI estimated using the estimation formula before local warming revealed that the bias and precision were −0.20 and 0.67 L min^−1^ m^−2^, respectively; the 95% limit of agreement was from −1.53 to 1.14 L min^−1^ m^−2^; and the percentage error was 62.7%. After warming, the bias and precision improved to −0.05 and 0.37 L min^−1^ m^−2^, respectively; the 95% limit of agreement narrowed from −0.78 to 0.68 L min^−1^ m^−2^; and the percentage error markedly improved to 34.3% (Figure 4).

## 4 DISCUSSION

In this study, we attempted to examine the feasibility of a new method for CI measurement by breath holding for 20 s and to identify the factors that affect LFCT measurement. The two study parts obtained the following findings:

1. LFCT measurement with the new method was successfully performed in most of the patients.
2. Low local temperature of the fingertip substantially increased LFCT, especially at <31°C.
3. It is highly possible that reliable LFCT values can be obtained by increasing the local temperature, such as that of the fingertip.
4. Direct correction by local warming along with mathematical correction might enable a simple and economical estimation of CI in most patients.

### 4.1 Data acquisition possibility with the new device

As we could obtain a clear peak in the LFCT curve of 45 of 53 patients (85.0%) in study 1 and in nine of 10 patients (90%) in study 2, we can conclude that the data acquisition rate of the new device was high enough to be used in the clinical setting. However, the fact that we excluded those who could not perform a breath hold, was under hemodialysis therapy, and have severe pulmonary diseases need to be mentioned. As overcoming this weak point is difficult at this time, this method should be used in a patient population similar to that used in this study at the beginning stage.

### 4.2 Safety of measuring LFCT

There were no patients who suffered from obvious discomfort or worsening original diseases after measuring LFCT. We consider that LFCT can be measured safely as long as physicians pay minimum attention to stable patients.

### 4.3 Determination of correction factors for CI measurement

Although we picked up 10 coefficients to find the top four parameters that had good correlation with CI, we cannot deny the possibility that parameters we did not measure in this study have a stronger correlation with CI. We have to search such parameters in the future study.

### 4.4 Effect of finger temperature on the CI‐1/LFCT relationship

In the whole patient group, the correlation between 1/LFCT and CI was weak (correlation coefficient, *r* = .51). However, when we focused on patients with finger temperature ≥31°C, the correlation coefficient markedly improved to *r* = .81 in study 1. To confirm the effect of low fingertip temperature, we performed actual warming of the forearm and wrist for validation in study 2. We found that the coefficient of the CI‐1/LFCT relationship improved from 0.69 to 0.82 by increasing the finger temperature, and this result indicated that keeping the finger temperature above a certain threshold is essential to obtain reliable LFCT values. With respect to the threshold temperature for measuring LFCT, we considered that 31°C might be the most suitable temperature judging from our results because in study 1, patients with finger temperature ≥31°C showed a consistent and clear negative CI‐1/LFCT correlation that was almost identical to that obtained in our previous study (Hosokawa et al., 2015) and warming to showed no LFCT decrease in study 2. Although we set the cut‐off value as 31°C, to find a more detailed cut‐off value we need more patients. Because fingertip temperatures were vulnerable below the first decimal point and there were patients number deviation among temperatures in this study, we could not verify the cut‐off values in more detail.

With respect to the close relationship between finger temperature and blood flow, previous studies have also reported similar results (Coffman, 1972; Nagasaka et al., 1985). The mechanism is considered as follows: Arteriovenous anastomoses are widely distributed subcutaneously at the extremities and are mainly controlled by sympathetic nerves (Walløe, 2016). A decrease in temperature causes a skin vasomotor response, which lowers blood flow in the hands and fingers through adrenergic nerve excitation to prevent heat dissipation. On the contrary, the blood flow volume in the hand is increased by indirectly warming the hand (Brajkovic & Ducharme, 1985). Thus, our observation that those who had lower fingertip temperatures had longer LFCT was not due to a decrease in CO but due to a decrease in blood flow caused by the constriction of peripheral blood vessels.

Concerning the question of whether LFCT might be decreased more by aggressive warming of the hand or forearm, we considered that once the temperature of the hand exceeds the threshold temperature, LFCT would remain at the point that is purely determined by CI. This is because we observed that the point to which LFCT moved after warming was the same point that could be expected using the relationship between CI and 1/LFCT in our previous study or in a study by another group (Kwon et al., 2016). Thus, we believe that warming the forearm and wrist to increase blood flow over a certain threshold would be essential for accurate LFCT measurement. This point was also highlighted by the Bland–Altman plot analysis in study 2, which showed that the percentage error of 62.7% between the measured and estimated CI before warming significantly improved to 34.3% after warming. We considered that although our method reached an almost practically sufficient level, it needs to be further refined to reduce the percentage error to <30% by taking other correction parameters into the calculation because the percentage error of a new method for estimating CI should be <30% according to a previous study (Critchley & Critchley, 1999).

Although when core temperature is increased, CO will increase because of reduction of peripheral vascular resistance (Wilson & Crandall, 2011), our method increased the finger temperature alone and did not change core temperature significantly. Although we measured a tympanic membrane temperature using a tympanic thermometer as body temperature before and after warming and we only noticed that the tympanic temperatures minimally increased. A review article about passive heat stress and cardiovascular system (Crandall & Wilson, 2015) shows that temperature change within the range of ≤2°C from normothermia has no direct effect on inotropes and heart rate increase. Thus, we regard that the effect of local warming on CO is very limited and negligible.

### 4.5 Effect of heart rhythm to CI‐1/LFCT relationship

The patients with atrial rhythm did not show the positive CI‐1/LFCT correlation. As for the reason why patients with atrial fibrillation rhythm did not show positive CI‐1/LFCT correlation, we consider that it would be the result of wide variability of stroke volume caused by heart rate dispersion of atrial fibrillation rhythm. We needed a larger number of atrial fibrillation patients to prove this point.

### 4.6 Utility and applicability to measure and to increase the fingertip temperature in the real clinical setting

In study 2, we warmed the wrist of one side by applying a heating pad for 10 min. We confirmed that all fingertip temperature became more than 31°C by this method without any skin disorders or worsening systemic condition. Although it would take some time to measure and warm the wrist (maximally, additional 15 min), it would be still simple and short time measurement which can be feasible in a clinical setting compared with other methods to measure CO like echocardiography or MRI.

### 4.7 Limitations

Our study had several limitations. First, this was a single‐institution analysis, and data were collected from a small number of subjects. In particular, only 10 subjects were enrolled in study 2 owing to time limitations. We plan to further increase the number of cases and study parameters as well as the coefficients in the next study. Another limitation was that this study did not adopt CI obtained by cardiac catheterization as a gold standard method. However, as the study also included patients without HF and healthy subjects, an invasive cardiac catheterization method could not be used. Finally, as warming the forearm and wrist routinely in the clinical setting would need additional time and effort, an easier to use and simpler warming device, or a mathematical method that can yield a more accurate result without warming, would be needed.

In this study, we did not analyze the effect of medication. Some medications like vasodilators, beta‐blockers, or other medications may affect CO. However, we believe that the relationship between CO and LFCT might be kept constant even under the influence of drugs, though not being exactly known. In the future study, we would like to analyze the effect of medication on LFCT.

In order to judge whether this device can be used in a clinical setting, it would be crucially important to evaluate the intrasubject variability and the interobserver variability of our methods. In current studies, the number of recording trials of LFCT varied from two to five times according to the patient. The deviation of LFCT values tended to be higher in the patients whose LFCT were recorded fewer times. This may mean that the patients who can easily repeat the measurement produce stable results. As for the interobserver variability, the source of variation is considered as how the observer orders to stop and restart breathing to the patients and how the observer decided the nadir of SpO_2_. As we had unified the protocol of stopping and restarting breathing in our study, we believe that there would be only small variability arising from this point. As for variation arising from deciding the nadir, we have noticed that nadir becomes double or even triple in some patients and, in another group, we could not decide a nadir at all. To cope with such patients, we are trying to make a criterion to decide to accept or to discard the nadir value. However, as the rate of such patients with undetermined nadir is small (<10%), the intraobserver variability as a whole must be small even at this point and it might be possible to make it smaller in near future.

## 5 CONCLUSION

We developed a new device for estimating CI by measuring LFCT after a brief breath hold in the awake state, and the measurement was feasible in 85%–90% of the cases. Our results suggested that a low fingertip temperature strongly and negatively influences the CI‐1/LFCT relationship, causing an error; however, increasing the local temperature can restore the original LFCT value and would provide an accurate estimation of CI especially when used together with a correction equation.

## DISCLOSURES

SA is receiving unrestricted funding from Philips‐Respironics Inc. and Teijin Home Healthcare.

## AUTHORS' CONTRIBUTIONS

T Tobushi was responsible for data acquisition from patients. This author takes responsibility for all aspects of the reliability and freedom from bias of the data presented and their discussed interpretation. KM, KS, and MA contributed to the development of devices and software for the team, and analyzed the data. All data were re‐reviewed by all authors. These authors take responsibility for all aspects of the reliability and freedom from bias of the data presented and their interpretation. KF analyzed the data of study 2 and conducted project management of study 2. T.Tohyama and MH advised the team about the statistical scheme and conducted project management of study 2. YY contributed to the final version of the manuscript. TK and RN supervised the cardiology team and provided clinical opinions. SA conceived the presented idea and led the team as the primary investigator. This author takes responsibility for all aspects of the reliability and freedom from bias of the data presented and their interpretation.

## TRIAL REGISTRATION

Study 1: UMIN‐CTR Clinical Trial: UMIN000030178

Study 2: Japan Registry of Clinical Trials ID; jRCTs072180003

## Supporting information



Figure S1a

Figure S1b
