# Supplementary figures and images for: Local temperature control improves the accuracy of cardiac output estimation using lung‐to‐finger circulation time after breath holding
Source: Physiol Rep. 2020 Nov 7;8(21):e14632. doi: 10.14814/phy2.14632 (PMC7648652; doi:10.14814/phy2.14632)

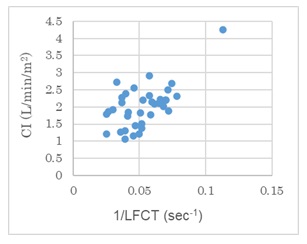

Supplement: Supplementary file 1 — Figure S1a [file PHY2-8-e14632-s001.jpg]

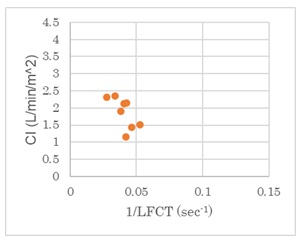

Supplement: Supplementary file 2 — Figure S1b [file PHY2-8-e14632-s002.jpg]
